# Characteristics of the Non-Isothermal and Isothermal Crystallization for the β Polymorph in PVDF by Fast Scanning Calorimetry

**DOI:** 10.3390/polym12112708

**Published:** 2020-11-16

**Authors:** Ernesto Pérez, Irene Angulo, Enrique Blázquez-Blázquez, María L. Cerrada

**Affiliations:** Instituto de Ciencia y Tecnología de Polímeros (ICTP-CSIC), Juan de la Cierva 3, 28006 Madrid, Spain; ernestop@ictp.csic.es (E.P.); ireneangulob@hotmail.com (I.A.); enrique.blazquez@csic.es (E.B.-B.)

**Keywords:** poly(vinyl fluoride), FSC, β and α polymorphs, isothermal and non-isothermal crystallization

## Abstract

Structuring at very high rates has become one of the current and important topics of interest in polymer science, because this is a common protocol in the processing of films or fibers with industrial applicability. This work presents the study by fast scanning calorimetry, FSC, of poly(vinylidene fluoride), paying special attention to the conditions for obtaining the β phase of this polymer, because it is the one technologically more interesting. The results indicate that this β phase of poly(vinylidene fluoride) is obtained when the sample is isothermally crystallized at temperatures below 60 °C. Under non-isothermal conditions, the β polymorph begins to be observed at rates above 400 °C/s, although a coexistence with the α modification is observed, so that exclusively the β phase is obtained only at rates higher than 3000 °C/s.

## 1. Introduction

The polymer industry seeks for materials with short processing times and, because a large number of polymers are semicrystalline [1], fast crystallizing polymers are preferable. Under those processing conditions, polymer crystallization cannot be considered an “equilibrium” phenomenon, because it is not feasible to separate the thermodynamics from kinetic effects on such processes. Although a complete understanding of the overall polymeric crystallization has not been attained, the general concept of the classical nucleation theory, suggesting the initial formation of stable crystal nuclei followed by their further growth, is still widely accepted. In fact, polymeric material crystallization is always limited by molecular mobility, and very often leads to metastable phases, which are considered as an intermediate stage in the polymer crystallization, as shown by Strobl [2,3]. Further evidences of the formation of metastable phases under drastic conditions (high cooling rates and/or high deformation rates) have been widely reported for isotactic polypropylene (iPP) [4,5,6,7], for polyamide 6 [8], and for polyethylene terephthalate (PET) [9].

Awareness of the relationships between those processing conditions and the structure derived from the materials obtained is a crucial point in polymers in order to achieve products matching the required properties. Therefore, an exhaustive structural characterization is mandatory to evaluate features of macrochains either within their amorphous state or crystalline regions (if any) together with knowledge of phase transitions. The investigations for studying crystallization and melting are usually performed using conventional experimental techniques such as X-ray scattering [10,11] or differential scanning calorimetry (DSC) [12,13]. Sometimes, these experiments do not allow analyzing the relevant processes with the required time resolution because their available cooling (or heating) rates are several orders of magnitude lower than the ones applied in industrial processes. Nevertheless, important progress has been made regarding the time resolution of X-ray scattering by using synchrotron radiation in combination with fast detectors. Concerning calorimetric analysis, a remarkable advance has taken place over the last twenty-five years, allowing achieving high and controlled scanning rates up to 10^6^ °C/s or even higher. Activities in the field of fast scanning chip calorimetry (FSC) on heating and cooling were successfully initiated by the works of Allen et al. [14,15,16].

Techniques capable of applying very fast rates are also a key tool for studying those systems with a polymorphic behavior where one or more of their crystalline lattices are exclusively developed under those rates. In addition to the polymers aforementioned, poly(vinylidene fluoride) (PVDF) is one of those systems. This polymer may present, depending on the crystallization conditions, five different crystal polymorphs: α, β, γ, δ and ε [17,18]. Three of them (α, β and γ) can be achieved during melt crystallization. At regular cooling rates, crystallization of either α or γ crystals can be developed, depending on temperature. The α phase is normally obtained by melt crystallization at temperatures below 160 °C. Temperatures above this value lead to a mixture of α and γ phases, the γ fraction being increased with crystallization temperature and time [19]. On the other hand, the β lattice, which has aroused the more technological interest because of its electroactive behavior, requires an effective high-rate quenching procedure to be attained from the melt [20]. The β modification in quenched samples coexists with the α form [20,21] and the ratio between both in the PVDF crystalline regions depends on the quenching rate.

Graddys et al. [22] showed for the first time that pure β phase in PVDF could be attained during crystallization from the melt at cooling rates above ca. 2000 K/s by using a self-made thin-film chip calorimeter. More recent research from this group [23], using a non-adiabatic thin-film chip calorimeter, applied cooling rates as high as 100,000 K/s, which were found to be sufficient to maintain the polymer in the amorphous state, and analyzed the response through isothermal tests within a temperature range from 76 to 125 °C. Another investigation [24] demonstrated that, by using commercial FSC, homogeneous crystal nucleation yielded dominant α-phase crystallites at cooling rates above 500 K/s, also finding a minor amount of β phase crystallites after long-term annealing of the fast-cooled PVDF at low temperatures. Later FSC experiments confirmed that the incorporation of poly(methyl methacrylate) (PMMA) into PVDF shifted the α to β crystal transition to lower cooling rates [25]. Moreover, several investigations have used FSC for the characterization of copolymers and terpolymers of PVDF [26,27,28].

The aim of this investigation was to gain a deeper understanding of the crystallization of the pure β polymorph in PVDF. For that, crystallization experiments performed under either non-isothermal or isothermal conditions from the melt were evaluated in a broad range of cooling rates and crystallization temperatures. The range of temperature in the isothermal tests for the β development was broader than the intervals used until in the literature [22,23], providing important information about the crystalline PVDF β phase. Other experiments using conventional DSC were carried out to complement the study, in addition to X-ray and FTIR measurements to show main structural differences between the α phase, which is thermodynamically the most stable crystalline form, and the β polymorph, which is, for this polymer, the most interesting from a technological standpoint.

## 2. Materials and Methods

### 2.1. Materials

A commercially available poly(vinylidene fluoride) (PVDF) with trade name of Kynar 741, kindly supplied by Arkema, was used in the present research. It was in a powder form.

### 2.2. Film Preparation

Films with thickness of around 100 microns were obtained by compression molding in a Collin press between hot plates (210 °C) at pressure of 2 MPa for 5 min. Three different thermal treatments along a cooling process were applied. The first one consisted of a slow cooling to room temperature at the inherent cooling rate of the press after the power was switched off (the cooling rate was around 2 °C/min). Pressure was maintained constant at 2.5 MPa for this treatment. This process was designated as S.

The second protocol involved a relatively fast cooling (around 100 °C/min) between plates of the press refrigerated with cold water from the melt to room temperature. This film was labeled as Q.

Finally, a section of the Q film was re-melted at 210 °C in a Mettler FP82HT hot stage, and rapidly quenched into a mixture of acetone and dry ice (film named as Q_CO2_).

### 2.3. X-ray Diffraction

Wide-angle X-ray diffraction (WAXD) patterns were recorded in the reflection mode by using a Bruker D8 Advance diffractometer provided with a PSD Vantec detector (from Bruker, Madison, WI, USA). Cu Kα radiation (λ = 0.1542 nm) was used, operating at 40 kV and 40 mA. The parallel beam optics was adjusted by a parabolic Göbel mirror with a horizontal grazing incidence Soller slit of 0.12° and a LiF monochromator. The equipment was calibrated with different standards: Al_2_O_3_ (Corundum) and Cr_2_O_3_. A step scanning mode was employed for the detector. The diffraction scans were collected with a 2θ step of 0.024° and 0.2 s per step.

### 2.4. Fourier Transform Infrared Spectroscopy

The different crystal lattices existing in the PVDF films were studied by Fourier transform infrared (FTIR) spectroscopy, over a wavenumber interval from 4000 to 650 cm^−1^, using a total attenuated reflectance device (FTIR-ATR). Spectra were recorded on a PerkinElmer Spectrum Two spectrophotometer with a resolution of 4 cm^−1^.

### 2.5. Differential Scanning Calorimetry (DSC)

Calorimetric analyses were performed in a TA Instruments Q100 calorimeter connected to a cooling system, under N_2_ atmosphere, and calibrated with different standards (indium for enthalpy, and zinc and indium for temperature). The sample weights were in the range from 6 to 8 mg. A temperature interval from −65 to 200 °C was studied at a scanning rate of 20 °C/min (0.333 °C/s).

### 2.6. Fast Scanning Chip Calorimetry (FSC)

A commercial power-compensation differential scanning chip calorimeter Flash DSC 1 from Mettler Toledo was used for the FSC analysis. Comprehensive details of this equipment can be found elsewhere [29]. By means of a two-stage intracooler device (Huber TC100), temperatures as low as around −100 °C could be reached. The samples were typically prepared in a first stage as films of 10–20 μm thick with a microtome. From those films, and with the aid of a microscope attached to the equipment, a small piece was cut with a scalpel, and placed in the middle of one of the two sensors of the calorimeter chip (previously calibrated with the parameters from the factory, and conditioned afterwards). Ideally, samples below around 50 ng were required in order to attain the maximum rates of the device (4000 °C/s on cooling and 40,000 °C/s on heating) without excessive problems of overheating.

The cooling rates used in the non-isothermal experiments ranged from 10 to 3000 °C/s. They were selected considering the mass of sample involved. The sample weight was estimated from the apparent enthalpy of melting obtained by FSC after cooling from the melt at the same rate as in conventional DSC (20 °C/min = 0.333 °C/s) by comparison with the actual enthalpy. It turned out to be 78 ng. Moreover, the peak melting temperature has been also considered for the FSC tests in what the initial temperature for the cooling experiments were set to a value around 60 °C above those melting temperatures.

Isothermal runs were also carried out at several temperatures, ranging from 40 to 135 °C.

## 3. Results and Discussion

### 3.1. Structural Characteristics

**Figure 1:**
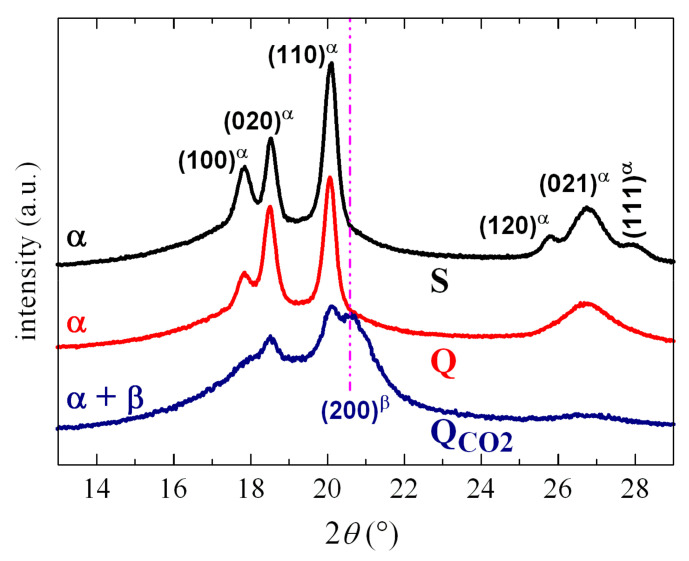
X-ray profiles for poly(vinylidene fluoride) (PVDF) films processed at different rates. They have been shifted for a better visualization.

Figure 1 shows the diffractograms for various PVDF samples. Samples S and Q (see Experimental) were obtained by compression molding and cooled at 2 and 100 °C/min, respectively. Both of them show the typical diffractions of the α phase, with the difference that the sample S, slowly cooled from the melt, exhibited narrower and better defined diffractions because processing conditions allowed the formation of thicker and more perfect crystals. This more favorable protocol was responsible for the proper resolution of the group of diffractions found between 26° and 28°.

The Q_CO2_ sample was rapidly cooled from the melt by immersion in acetone/dry ice. Its profile compared with those for the S and Q films showed important differences. The (100), (020) and (110) reflections from the α crystals became broadened and their intensity was significantly diminished. Furthermore, the group of diffractions located between 26° and 28° almost disappeared and a new reflection was seen at about 20.6°. This is associated with the presence of β crystallites, which coexist with the α ones. The processing protocol used allows the development of both polymorphs. An approximate calculation identified that the α/β ratio was around 50%, from the deconvolution of the diffractograms, including the amorphous halo.

Although the specific cooling rate in this Q_CO2_ sample was not known, it was estimated that it could range between 170 and 500 °C/s, based on information derived from the industrial production of films and fibers crystallized by immersion in thermostatized baths. However, even at such high cooling rates, the PVDF β phase was not the only one achieved in the specimen labeled as Q_CO2_, and its crystallites coexisted with other α crystals, as clearly noticed in Figure 1.

**Figure 2:**
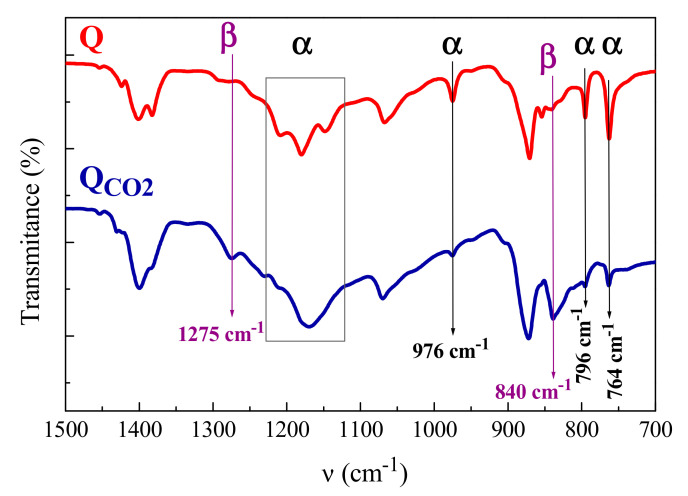
FTIR spectra for PVDF films processed at two different rates. Characteristic bands have been identified in this spectral region. They have been shifted for a better visualization.

Figure 2 represents the FTIR spectra for the Q and Q_CO2_ samples; this technique turned out to be a very useful tool for identifying the most common polymorphs in PVDF (α, β, and γ), because each one showed characteristic bands [19,30,31], as listed in Table 1.

The Q sample exhibited only the specific bands for the α crystalline lattice, while the particular bands corresponding to the β polymorph were also observed in the Q_CO2_ specimen. Moreover, a noticeable decrease in intensity of the α bands was found in the latter because its amount was significantly reduced. The cooling protocol applied here seemed to be more efficient than that used previously [24], where fast-cooled films were prepared by quenching into liquid nitrogen followed by a slow heating back to room temperature. All of these results are in a perfect agreement with those derived from X-ray diffraction.

### 3.2. Non-Isothermal Crystallization Experiments

**Figure 3:**
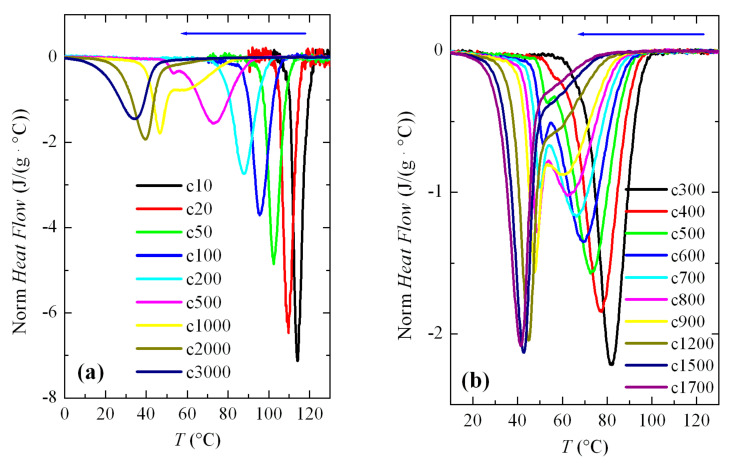
Fast scanning calorimetry (FSC) cooling curves (normalized to the weight of sample and cooling rate) corresponding to PVDF cooled from the melt at the rate indicated (cx, x in °C/s): (**a**) a global cooling rate interval; and, (**b**) a range of rates where the two exothermic processes clearly overlapped.

In order to investigate the formation of the β modification in PVDF, various cooling–melting experiments were performed. Figure 3a shows a series of FSC curves (normalized to sample weight and cooling rate) from cooling the PVDF sample at a wide range of rates, between 10 and 3000 °C/s. Two aspects are remarkable from Figure 3a. Firstly, the signal-to-noise ratio deteriorated greatly at rates below 50 °C/s, although curves are perfectly quantifiable. Secondly, and more importantly, a second crystallization exotherm begins to appear at rates above 500 °C/s in the region of low temperatures, together with that observed at higher temperatures.

Intensity and width of this crystallization appearing at low temperatures increases as the cooling rate does. Thus, it starts just as a shoulder coexisting with a main exotherm located at higher temperature (see Figure 3b), and becomes the only exothermic process observed at the rate of 3000 °C/s, as noticeable in Figure 3a. Both peaks were moved to lower temperatures with an increasing cooling rate, their dependence on rate being smaller for that appearing at low temperatures. The high temperature exotherm was associated with crystallization of the α polymorph, while the peak at low temperature was ascribed to the formation of β crystals, as reported [22,23,24,25].

Figure 3b displays in more detail the region at rates ranging between 300 and 1700 °C/s where these two crystallization processes, formation of the α and β phases, coexist. It was observed that a small but appreciable amount of β phase was already detected at 400 °C/s. At higher rates, this exotherm was a very narrow peak with a low intensity. If the rate was raised further, the peak related to the α form began to involve less and less enthalpy and was transformed into a shoulder at the right temperature side of the β crystallization, i.e., the α crystallization becomes the secondary exotherm. These results agree rather well with those obtained by other groups [22,23,24,25]. Some differences found can be attributed to the distinct equipment used, self-made versus commercial calorimeter, as well as variations in the weight of the PVDF samples. In fact, cooling experiments performed at similar rates show lower crystallization temperatures, *T_c_*, in our research compared with those for the specimen reported, for instance in [22]. The weight of that sample was 460 ng and ours weighed 78 ng.

**Figure 4:**
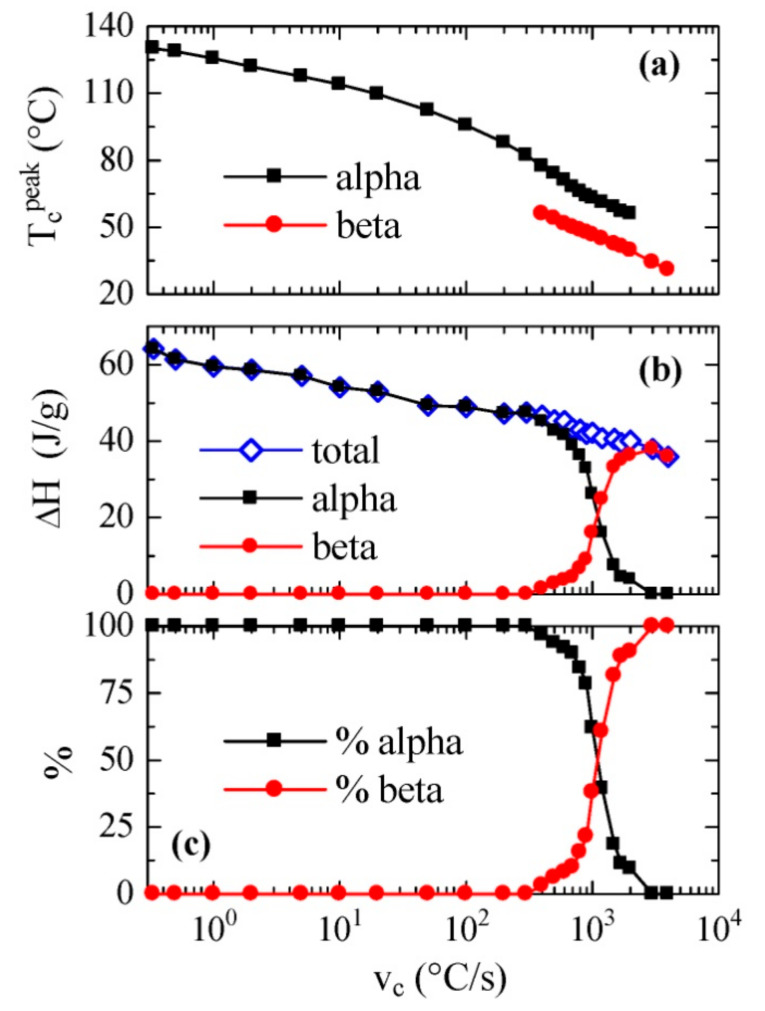
Dependence on cooling rate of: (**a**) peak crystallization temperatures; (**b**) enthalpies; and, (**c**) percentage of each polymorph deduced, from FSC experiments for PVDF cooled from the melt.

All the cooling curves represented in Figure 3, together with some others performed using FSC but at much smaller rates (not shown), typical of conventional DSC, have been analyzed and the values of the crystallization peak or peaks and their corresponding enthalpies have been achieved. The results are depicted on a logarithmic scale in Figure 4 as a function of the cooling rate. Figure 4a shows the temperature variation of the crystallization exotherm or exotherms. It is noticed that a fairly linear variation is observed for the low temperature exotherm, assigned to the β phase, with values of peak crystallization temperatures ranging between approximately 60 and 30 °C with a decreasing cooling rate. This trend is somewhat different to those described previously [22,23], because a constant value was reached at either the high or low temperatures of the rate interval, respectively. Nevertheless, it is analogous with that shown for neat PVDF in [25], where commercial equipment was also used.

On the other hand, variation in the peak temperature corresponding to the α phase was almost parallel along the coexistence zone, although this exotherm was detected at temperatures approximately 15–20 °C higher than those observed for the β phase.

Figure 4b shows the dependence on cooling rate of both the total enthalpy of crystallization and the enthalpy of each of its two components. It is noticeable that the total enthalpy exhibited a continuous decrease as cooling rate increased, without very evident discontinuity when the β modification was formed. This means that there were not very significant differences between the enthalpies of both polymorphs α and β. Furthermore, the β form began to appear at rates greater than 400 °C/s, and its enthalpy proportion clearly increased progressively with the cooling rate.

The values found for the α and β polymorphs are in agreement with those previously reported for neat PVDF [25] but they are significantly higher than those obtained previously using a self-made thin-film chip calorimeter [22,23].

The percentage of each phase as a function of rate is represented in Figure 4c. It is remarkable that the 50% of each one of them was obtained at an approximate speed of 1100 °C/s, while, as mentioned, the α phase was only obtained at rates below 400 °C/s, and only the β polymorph was observed at rates above 3000 °C/s.

**Figure 5:**
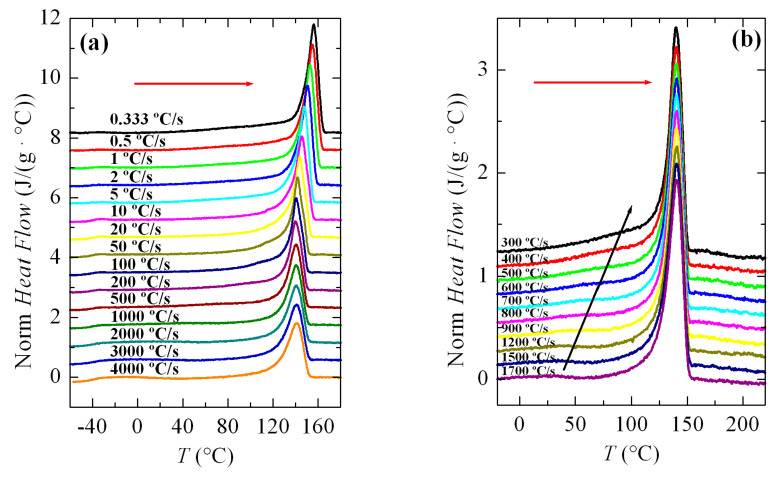
FSC melting curves at 500 °C/s (normalized to the weight of sample and heating rate) corresponding to PVDF cooled from the melt at the rate indicated (cx, x in °C/s): (**a**) a global cooling rate interval; and, (**b**) a range of rates where the two exothermic processes clearly overlapped on cooling. Curves have been shifted for the sake of clarity.

The melting processes after those crystallization experiments represented in Figure 3 are shown in Figure 5. Figure 5a shows that the main melting temperature, *T_m_*, remained practically constant between rates from 4000 to 100 °C/s, and there was only a clear dependence of the melting temperature on cooling rate below about 50 °C/s. This tendency seems to indicate that the constancy in the maximum temperature of the main endotherm at high rates is due to recrystallization phenomena. We will return to this topic later.

Another aspect to point out in Figure 5a is the observation of the glass transition temperature, *T_g_*, at around −40 °C, which is especially noticeable at higher rates.

Figure 5b shows the melting curves run at 500 °C/s for specimens crystallized at rates where both α and β polymorphs were developed. In this interval, the main melting point remained unchanged with the cooling rate. Nevertheless, because this figure is more enlarged than Figure 5a, a small endotherm (indicated by the black arrow) was observed, which would correspond to the melting-recrystallization of the crystals initially formed at those high rates. Accordingly, the main endotherm was ascribed to the melting of those recrystallized entities but not from the original ones. If the arrow was followed, a dependence on cooling rate would be observed for the initial melting process.

### 3.3. Isothermal Crystallization Experiments

One of the fundamental premises for studying isothermal crystallization is that cooling from the melt to the crystallization temperature must be fast enough to avoid crystallization on cooling down. Considering the limit for cooling rates in the equipment used (maximum cooling rate is 4000 °C/s), it was decided that a rate of 3000 °C/s would be applied for carrying out these experiments. Taking into account these considerations, it follows that the minimum crystallization temperature that could be analyzed was around 40 °C (see below). Nevertheless, there is no information of PVDF isothermal crystallization performed at this temperature, because the isothermal experiments reported in the literature for this polymer were carried out at various temperatures ranging from 60 to 180 °C [22,23].

Regarding the upper limit for the isothermal experiments, it must be remembered that crystallization rate is very slow at temperatures close to *T_m_*, so there will be a practical compromise limit between the total crystallization time and the signal-to-noise ratio for the corresponding isotherm.

**Figure 6:**
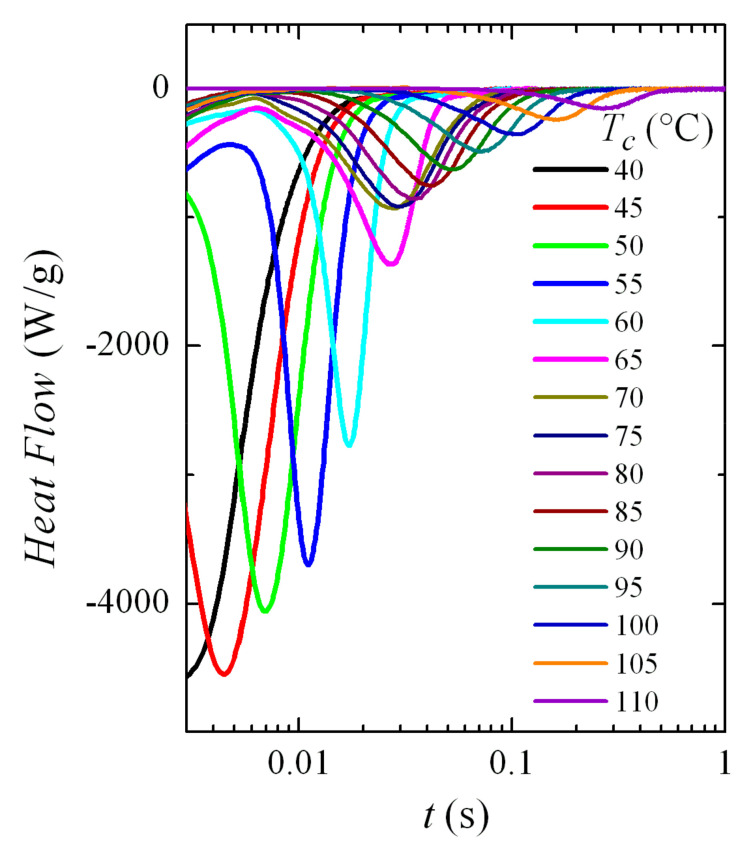
Normalized FSC crystallization isotherms (normalized to the weight of sample) obtained for PVDF in isothermal experiments at the indicated temperatures.

Therefore, the crystallization isotherms of PVDF in the temperature range from 40 to 110 °C have been analyzed by FSC. The corresponding isotherms can be seen in Figure 6. Despite the fact that part of the sample was crystallized at 40 °C before isothermal equilibrium, three distinct regions are clearly observed in this figure.

The first one takes place up to a *T_c_* of approximately 60 °C and crystallization time increases exponentially with temperature. Later, an intermediate zone is noticed in which a change of trend is observed in the isotherm curves, approximately between 65 and 75 °C; and, at higher temperatures, a third region is deduced where again an exponential increase in the crystallization time is observed with *T_c_*. Considering the previous experiments, the most feasible explanation is that the first region concerns exclusively the formation of the β phase, while the α crystals will be obtained in the third region; coexistence of both polymorphs is presumed in the intermediate region.

These intervals are coincident in certain ways with those derived from the non-isothermal crystallization results (see Figure 3 and Figure 4). It was found that the maximum temperature at which the exotherm corresponding to the β phase appeared was precisely 60 °C. However, that bimodal behavior, ascribed to the presence of both the α and β phases, is now not observed in the isotherms. Consequently, evaluation of the percentage for each phase was not possible in this temperature interval of eventual coexistence of both polymorphs for these isothermal experiments.

An approximate description of the crystallization kinetics has been carried out by means of the Avrami theories [32,33,34], widely used for describing the crystallization kinetics of polymers, despite the fact that this model was developed for metals. Analysis of the kinetics was carried out with stationary Avrami equation [32,33,34]:

(1) $$X_{C}(t)=1-\exp[-Kt^{n}]$$

where *X_C_*(*t*) is the material weight fraction that crystallizes at time *t*; *K* is the rate constant, where terms dependent on temperature are included together with information related to diffusion and nucleation rates; and, *n* is the Avrami exponent, which is a constant involving the types of processes that take place during nucleation and growth.

Determination of all of these parameters was carried out by conversion of the Avrami equation (numbered as (1)) into a double logarithmic form:

(2) $$\ln[-\ln(1-X_{C}(t))]=\ln K(T)-n\ln t$$

**Figure 7:**
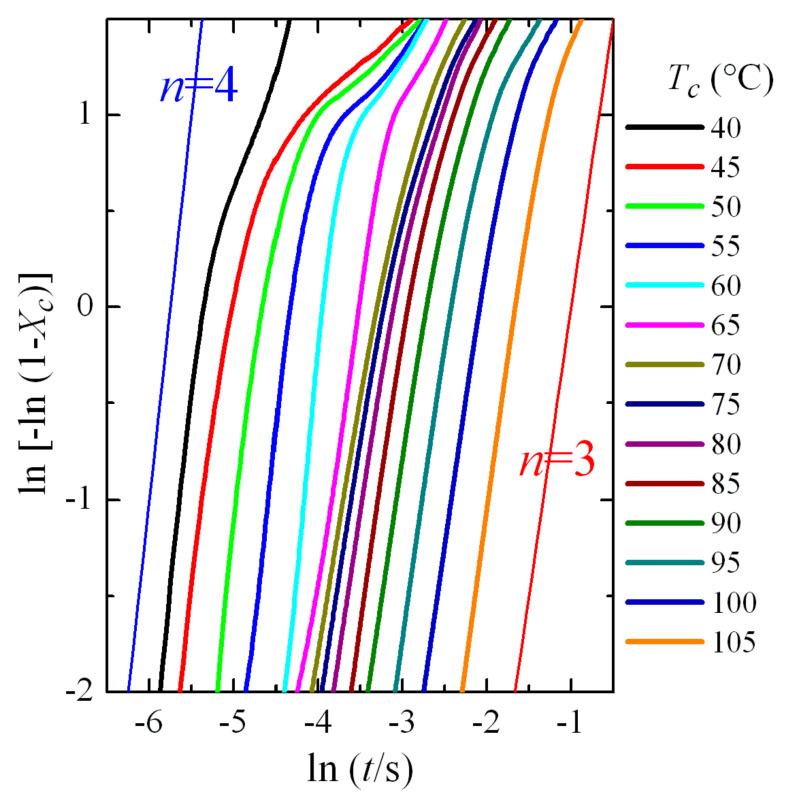
Avrami double logarithmic plot for PVDF at the indicated isothermal crystallization temperatures.

This Avrami double logarithmic representation is depicted in Figure 7. The aforementioned three regions are more clearly noticeable. Furthermore, a change in the Avrami exponent is also deduced, which shifts from being *n* = 4 at low crystallization temperatures to *n* = 3 at the highest ones. These values seem to indicate that, assuming three-dimensional growth, the process passes from homogeneous nucleation at low crystallization temperatures (formation of the β modification) to heterogeneous nucleation at high temperatures (polymorph α). We will see later in more detail the analysis of the Avrami parameters as a function of the crystallization temperature and the crystal modification obtained.

**Figure 8:**
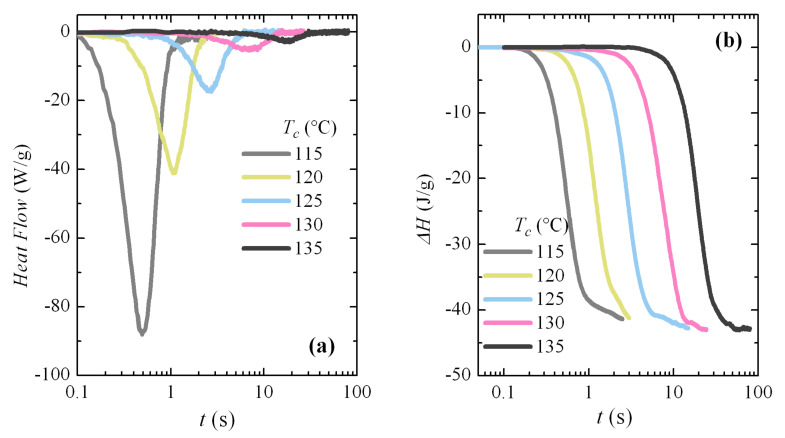
(**a**) Normalized FSC crystallization isotherms (normalized to the weight of sample) for PVDF at the indicated temperatures. (**b**) Variation of enthalpy with time for the indicated crystallization temperatures.

**Figure 9:**
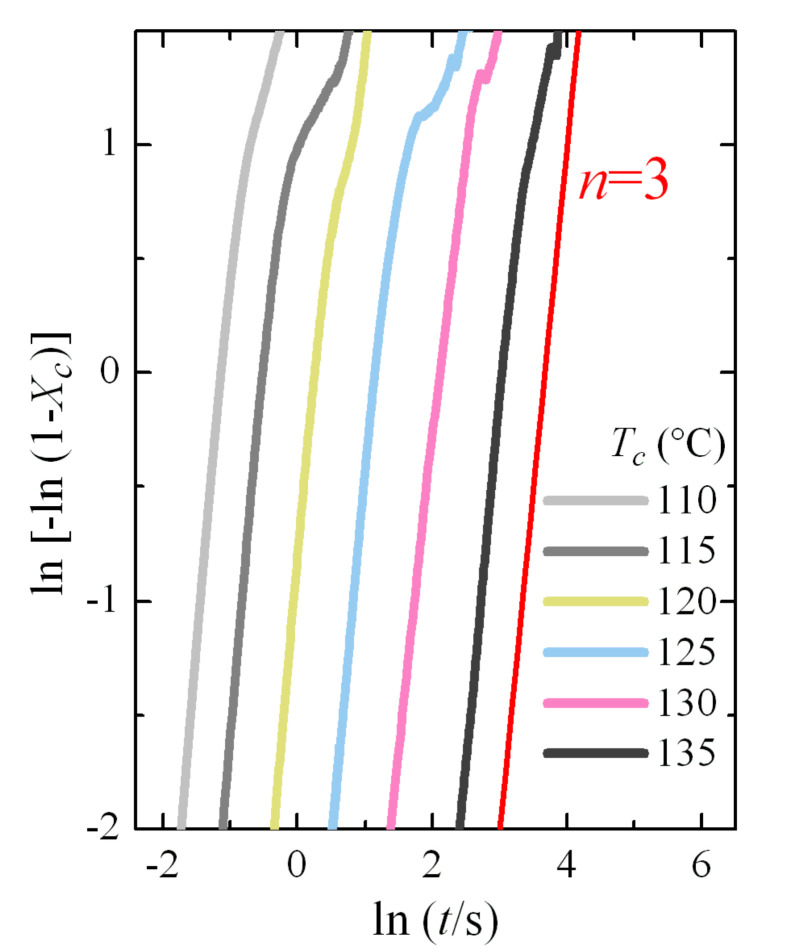
Avrami double logarithmic plot for PVDF at the indicated crystallization temperatures.

Additional isothermal crystallization experiments have been carried out at higher temperatures. A progressive deterioration for the signal-to-noise ratio was expected with decreasing crystallization rates, as seen in Figure 8a. However, independently of the increase in noise, the curves can be analyzed with relative simplicity; for instance, Figure 8b shows the variation in enthalpy with crystallization time for these isotherms. The typical sigmoidal shape is observed and the increase in the induction period is clearly deduced from them. The noise is especially evident at the highest times for each isotherm. The Avrami double logarithmic plot for these crystallization isotherms is shown in Figure 9. As expected, the Avrami exponent is close to *n* = 3 in this temperature range, where only the α modification is formed.

**Figure 10:**
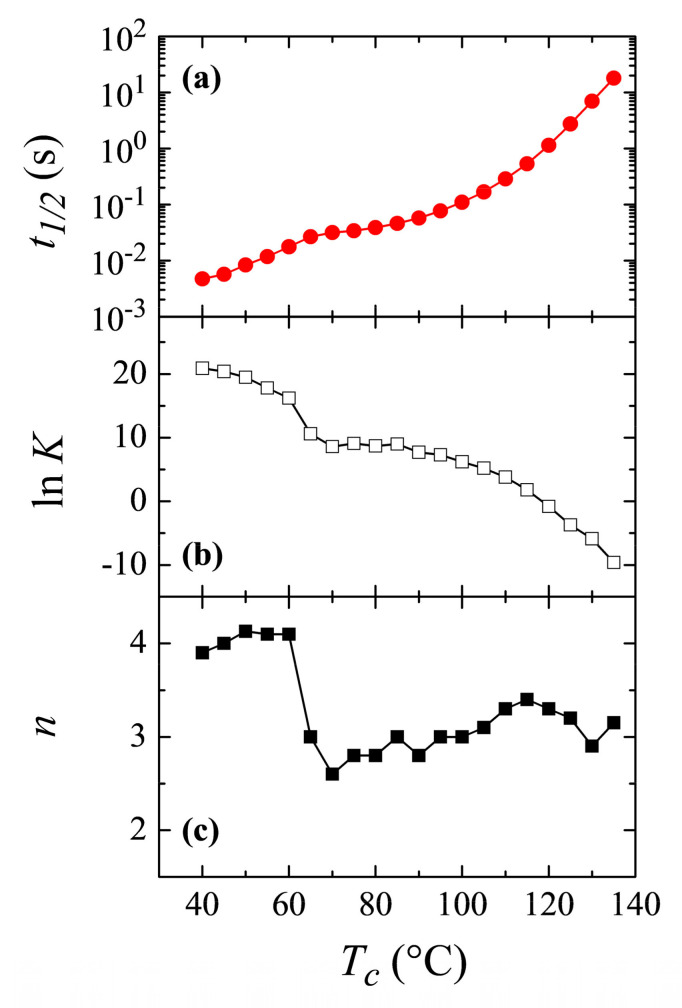
Dependence on crystallization temperature of: (**a**) the time to reach 50% of transformation, *t_1/2_*; (**b**) the Avrami rate constant; and, (**c**) the Avrami parameter.

The Avrami parameters obtained from all the above crystallization isotherms are presented in Figure 10b,c, together with the time required to reach 50% of the transformation, *t*_1/2_ (Figure 10a), as a function of the crystallization temperature. Both *t*_1/2_ and *K* constitute a measure of the rate of the process (actually the inverse of *t*_1/2_ represents the crystallization rate), while the exponent *n* is related to the nucleation and growth mode, as has been commented.

Crystallization rate exhibits a clear inflection point at temperatures around 60–65 °C, with higher rates in the region of low temperatures. It follows, evidently, that crystallization rate for the β modification is faster than that for the α polymorph and, because of this reason, the β form is the one achieved at low temperatures, being kinetically favored at those temperatures.

Regarding variation of the Avrami *n* exponent, Figure 10c clearly shows what was deduced and commented above: a change in the Avrami exponent is noticed. It takes the approximate value of 4 at low temperatures, exhibits a clear discontinuity at the temperature interval of 60–65 °C, and finally reaching values of around *n* = 3 at higher temperatures. Therefore, the change in the nucleation/growth mode discussed above is clear, indicating, most probably, the transformation from a homogeneous nucleation at low crystallization temperatures, where formation of the β modification occurs, to a heterogeneous nucleation at high temperatures where polymorph α is attained. It has to be considered, however, the aforementioned problem of applicability of the Avrami model for the crystallization of polymers, so it is not straightforward to deduce a physical meaning of the Avrami exponent, and a change in its value indicates mainly that the crystallization does not obey to a single-step equation. Anyway, the clear discontinuities observed in Figure 10 are, no doubt, indicative of the temperature interval where the formation of either the α or β polymorphs occurs.

Bimodality, derived from a possible coexistence of both the α and β phases, is not noticed in the crystallization isotherms. From the variation of Avrami exponent, seen in Figure 10c, it seems to be deduced that coexistence of the two polymorphs only occurs in a narrow range of temperatures, between 60 and 70 °C in isothermal crystallization experiments.

**Figure 11:**
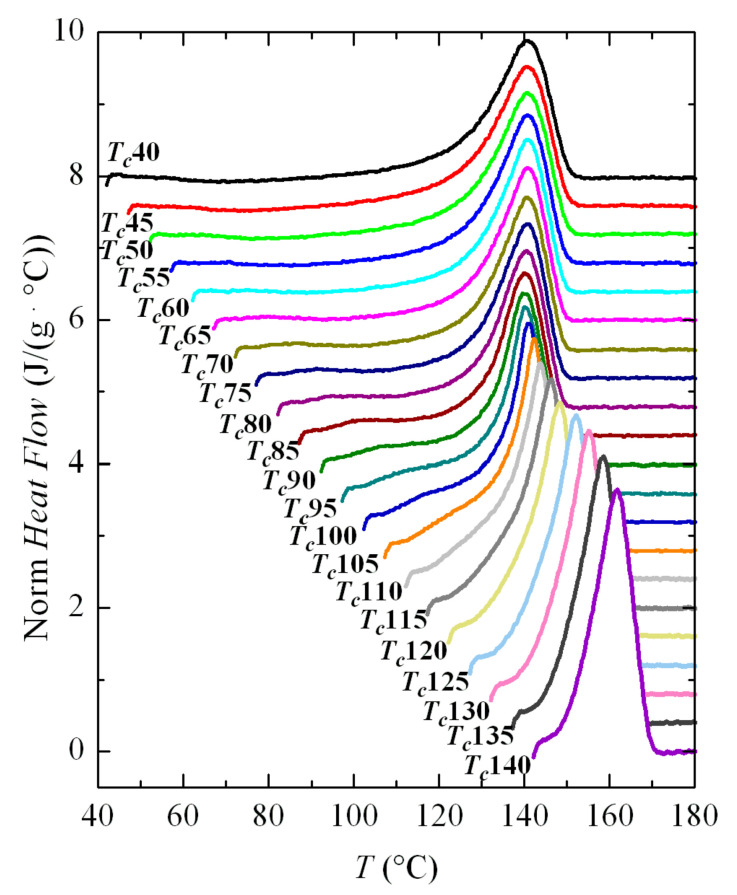
FSC melting curves at 500 °C/s (normalized to the weight of sample and heating rate) of the isothermally crystallized PVDF at the indicated temperatures. For clarity, the curves have been shifted vertically.

Melting curves after these crystallizations are depicted in Figure 11. As in the case of non-isothermal cooling, the main melting endotherm remains unchanged at low crystallization temperatures, and only a clear dependence of *T_m_* on *T_c_* is observed for the *T_c_* values above around 100 °C. The interpretation is again that there is a rapid process of fusion-recrystallization of the initial crystallites, which are very imperfect, so the main endotherm will correspond to the fusion of these recrystallized entities, and not the original ones. That occurs at the actual melting rate used (500 °C/s).

Higher melting rates have been tested, but it was not possible to avoid recrystallization and, therefore, observation of the true melting endotherm of these very imperfect crystallites did not become available in the FSC experiments performed because of limitations of the equipment. Unfortunately, the region in which these features occur completely involves the entire temperature range in which the β modification is developed, so it was not possible to attain information about the true melting temperatures of these β crystallites.

**Figure 12:**
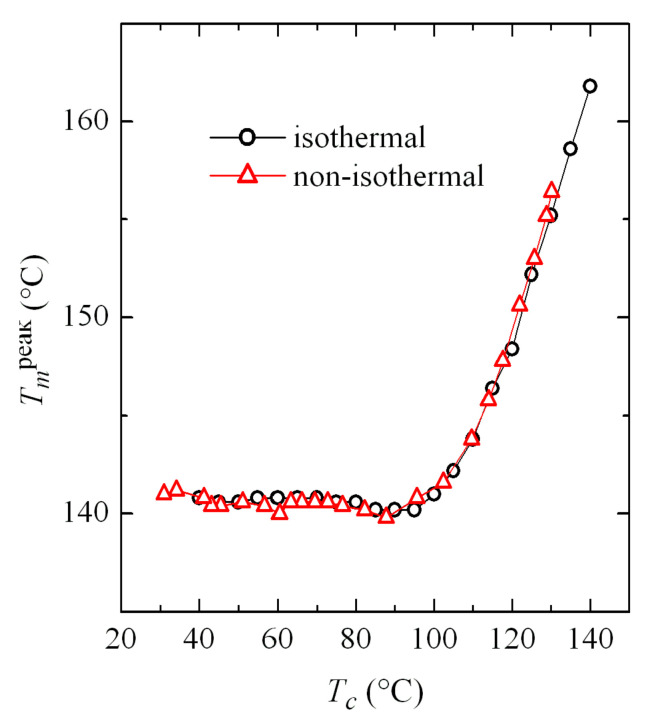
Variation of the melting temperature of the main endotherm with the crystallization temperature (see text to know the values taken for *T_c_* in non-isothermal tests).

Comparison of the results obtained in both isothermal and non-isothermal crystallizations turns out very interesting. Figure 12 shows the dependence on crystallization temperature of the melting temperature from the main endotherm (see Figure 5 and Figure 11). This crystallization temperature is obvious in the case of isothermal experiments. However, it is not so simple for non-isothermal tests, because the sample actually crystallizes in a wide range of temperatures. Accordingly, *T_c_* has been assigned as the most probable value defined by the minimum of the crystallization exotherm peak. However, there is a relatively broad temperature interval in which the α and β phases coexist, as observed in Figure 4a, where the variation of *T_c_* with cooling rate was represented. In those cases, a weighted average can be taken as the value of *T_c_*, considering the percentage obtained for each phase, which also appeared in Figure 4c.

Results represented in Figure 12 show an excellent agreement for the values obtained in both types of crystallization, which allows the extrapolation of information between both kinds of tests: isothermal and non-isothermal.

Isothermal crystallization of PVDF has also been studied by conventional DSC. Obviously, the available cooling rates are much slower in DSC than in FSC and, accordingly, the accessible range of crystallization temperatures is restricted to a much smaller range, and also in the high temperature zone, which implies that only crystallization of the α phase will be able to be observed. However, considering that the sample mass in DSC is several orders of magnitude greater than in FSC, the upper limit of crystallization temperatures achievable by DSC is noticeably higher.

**Figure 13:**
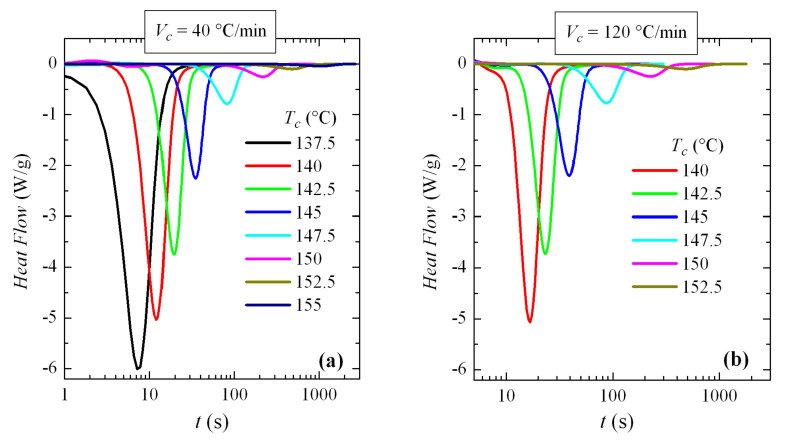
Isotherms of crystallization isotherms (normalized to the weight of sample), obtained by differential scanning calorimetry (DSC), for PVDF at the indicated temperatures and at cooling rate from the melt of: (**a**) 40 and (**b**) 120 °C/min.

Thus, the crystallization isotherms of PVDF over the available range of crystallization temperatures have been analyzed. For this, two different cooling rates from the melt have been used: 40 and 120 °C/min (remember, again, that the rates are usually expressed in °C/min in conventional DSC and in °C/s for FSC). The corresponding isotherms can be seen in Figure 13. An exponential increase in crystallization time with *T_c_* is observed, without any apparent discontinuity.

These isotherms were then analyzed. The variation of enthalpy with crystallization time exhibited for all of them a common sigmoidal shape (similar to those depicted in Figure 8b) and the exponent deduced from Avrami’s double logarithmic representation was of around 3. This was the expected behavior, because these experiments were performed at high temperatures where only the α modification was generated.

**Figure 14:**
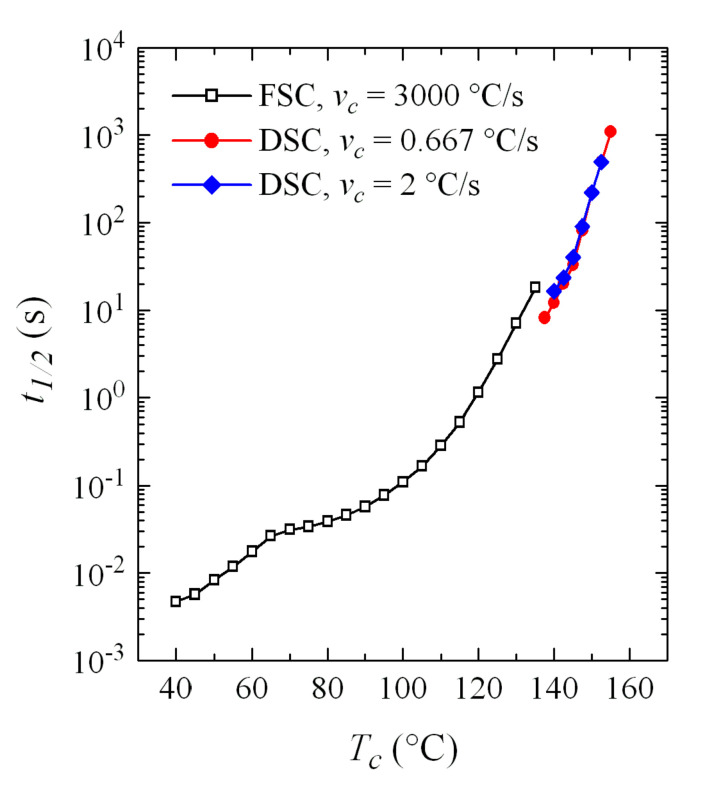
Comparison of variation with crystallization temperature of the time necessary to reach 50% of the transformation, *t*_1/2_, for the experiments of FSC and DSC.

Time to reach 50% of the transformation, *t*_1/2_, has also been determined, with the represented in Figure 14, compared with those obtained by FSC. It is observed that the agreement is outstanding, especially if the great difference in cooling rates from the melt is taken into account. In fact, and focusing on the DSC experiments, a small but evident increase in the *t*_1/2_ values was observed with the cooling rate: the data at 120 °C/min (2 °C/s) are slightly above those corresponding to cooling at 40 °C/min (0.667 °C/s). It is not surprising, therefore, that the *t*_1/2_ values for FSC, with a cooling rate of 3000 °C/s, are slightly higher than those of DSC.

## 4. Conclusions

The fast differential calorimetry technique, FSC, has proven to be a very useful tool to obtain, in real time, specific information about the formation of the β phase of PVDF by cooling from the melt at very fast rates.

The non-isothermal crystallization results from the melt indicate that at cooling rates above 400 °C/s, a second crystallization exotherm begins to appear as a shoulder in the low-temperature interval. Its relative importance becomes greater and greater as cooling rate increases. Thus, this low temperature exotherm was the only one observed at a rate of 3000 °C/s or higher. The α polymorph only crystallizes below 400 °C/s while the β modification begins to be developed at that rate and faster.

The 50% amount of each phase was obtained at an approximate rate of 1100 °C/s from the variation of the percentage of each phase as a function of cooling rate. Furthermore, the variation of the crystallization temperature for the β phase ran approximately parallel to that for the α modification along the coexistence zone, albeit with temperatures that were approximately 15–20 °C lower for the β phase.

The subsequent melting processes indicated that the main endotherm remains practically constant at the crystallization rates ranged from 4000 to 100 °C/s, and only a clear dependence on cooling rate of the melting temperature is observed below about 50 °C/s. This consistency with crystallization rates is assumed to be due to recrystallization phenomena. It is possible also to observe a small endotherm prior to the main melting peak, which would correspond to the melting-recrystallization of the initial defective crystals.

Crystallization isotherms for evaluation of the β polymorph formation have also been analyzed in the temperature range from 40 and 110 °C. These isotherms showed three distinct regions: up to a *T_c_* of approximately 60 °C, the crystallization time increases exponentially with temperature, corresponding to the formation of the β phase; afterward, an intermediate zone is seen in which a change of trend in the isotherm curves is observed, approximately between 65 and 75 °C, where both crystalline modifications coexist; and, a third region is observed (again with an exponential increase in crystallization time with the *T_c_*) where only α crystals are developed.

Avrami treatment pointed out a change in the Avrami exponent, which shifts from *n* = 4 at low crystallization temperatures to *n* = 3 at the higher ones. These values seem to indicate a transformation from a homogeneous nucleation at low crystallization temperatures, i.e., during formation of the β modification, to a heterogeneous nucleation at high temperatures when polymorph α is crystallized. Furthermore, the rate of crystallization exhibited a clear change in trend at temperatures around 60–65 °C, with higher rates at low temperatures, indicating that the crystallization rates for the β modification are obviously higher than those of the α polymorph, i.e., it is kinetically favored.

Melting curves obtained after isothermal crystallizations show, similarly to the non-isothermal experiments, that the main melting endotherm remains unchanged at low crystallization temperatures, and only for *T_c_* values above about 100 °C a clear dependence of *T_m_* with *T_c_* is observed.

Isothermal crystallization of PVDF has also been studied by conventional DSC. Comparison of the values obtained from both techniques for the time to reach 50% of the transformation, *t*_1/2_, allows observing an excellent agreement, especially if the great difference in cooling rates from the melt to attain the crystallization temperature is taken into account.

## Figures and Tables

**Table 1:** Characteristic bands of different PVDF polymorphs.

	α Form	β Form	γ Form
**Wavenumber** (cm^−1^)	408; 532; 614; 764; 796; 855; 976	445; 510; 840; 1275	431; 512; 776; 812; 1220
